# Mechanism of GLP-1 Receptor Agonists-Mediated Attenuation of Palmitic Acid-Induced Lipotoxicity in L6 Myoblasts

**DOI:** 10.1155/2022/6237405

**Published:** 2022-12-28

**Authors:** Mo-wei Kong, Yu Gao, Yu-yu Xie, En-hong Xing, Li-xin Sun, Hui-juan Ma, Han-ying Xing

**Affiliations:** ^1^Department of Endocrinology, Affiliated Hospital of Chengde Medical University, 36 Nanyingzi Street, Shuangqiao District, Chengde City, Hebei Province, China; ^2^Department of Dermatology, Chengdu Fifth People's Hospital, 33 Ma Shi Street, Wenjiang District, Chengdu City, Sichuan Province, China; ^3^Department of Endocrinology, Hebei Provincial People's Hospitall, No. 348, Heping West Road, Xinhua District, Shijiazhuang City, Hebei Province, China

## Abstract

**Methods:**

Cells were divided into 5 groups—control, high-fat, 10 nmol/L LR + 0.6 mmol/L palmitic acid (PA) (10LR), 100 nmol/L LR + 0.6 mmol/L PA (100LR), and 1000 nmol/L LR + 0.6 mmol/L PA (1000LR). CCK-8 method to detect cell viability, GPO-PAP enzymatic method to detect intracellular triglyceride content, and reverse transcription quantitative real-time polymerase chain reaction (RT-qPCR) and western blotting methods to detect fatty acid translocase CD36 (FAT/CD36) and fatty acid binding protein 4 (FABP4) in L6 cells, glucose-regulated protein 78 (GRP78), glucose transporter 4 (GLUT4) expression at the mRNA and protein levels, respectively, were performed.

**Results:**

We found that after PA intervention for 24 h, the cell viability decreased significantly; the cell viability of the LR group was higher than that of the high-fat group (*P* < 0.01). After PA intervention, compared with those in the high-fat group, GRP-78, FAT/CD36, FABP4 mRNA ((4.36 ± 0.32 vs. 8.15 ± 0.35); (1.00 ± 0.04 vs. 2.46 ± 0.08); (2.88 ± 0.55 vs. 8.29 ± 0.52), *P* < 0.01) and protein ((3338.13 ± 333.15 vs. 4963.98 ± 277.29); (1978.85 ± 124.24 vs. 2676.07 ± 100.64); (3372.00 ± 219.84 vs. 6083.20 ± 284.70), both *P* < 0.01) expression decreased in the LR group. The expression levels of GLUT4 mRNA ((0.75 ± 0.04 vs. 0.34 ± 0.03), *P* < 0.01) and protein ((3443.71 ± 191.89 vs. 2137.79 ± 118.75), *P* < 0.01) increased.

**Conclusion:**

Therefore, we conclude that LR can reverse PA-induced cell inactivation and lipid deposition, which may be related to the change in GRP-78, FAT/CD36, FABP4, GLUT4, and other factors.

## 1. Introduction

Insulin resistance (IR) is one of the pathologies of many metabolic diseases, including type 2 diabetes and obesity. Previous studies have shown that an increase in free fatty acids induces ectopic lipid deposition through multiple pathways and further leads to IR [1–3]. Glucagon-like peptide-1 (GLP-1), a hormone secreted by intestinal L6 cells, can promote insulin secretion by pancreatic B cells and has an extrapancreatic hypoglycemic effect. Liraglutide (LR) is a GLP-1 analogue with wide clinical use. Recent studies have shown that ppar gamma liraglutide exerts a protective effect by alleviating nonalcoholic steatosis and drug-induced steatosis [4]. Another clinical study also showed that LR has a good therapeutic effect in overweight, obese and type 2 diabetic patients [5]. However, the mechanism of action of LR still needs to be further explored.

Fatty acid translocase (FAT/CD36) is a fatty acid transport protein that can promote intracellular fat deposition. LR can improve cardiac diastolic dysfunction in patients with type 2 diabetes by affecting the expression of cardiac FAT/CD36 and converting fatty acids in cardiomyocytes into glucose [6]. The overexpression of adipocyte fatty acid binding protein 4 (FABP4) can promote fatty acid transport. Glucose-regulated protein 78 (GRP78) is a molecular chaperone of the endoplasmic reticulum and a classic marker protein of endoplasmic reticulum stress (ERS) [7]. Glucose transporter 4 (GLUT4) is a protein responsible for glucose transport across the membrane in eukaryotic cells, which mainly plays a role after insulin stimulation. When cells are in a state of IR, GLUT4 abundance usually decreases, making GLUT4 a useful indicator of IR.

Palmitic acid (PA) is a saturated fatty acid and one of the most common free fatty acids. Previous studies have found that PA-induced cellular IR is related to lipid deposition and ERS [8, 9]. Whether LR reduces skeletal muscle lipid deposition and ERS by affecting skeletal muscle fatty acid transport is unclear. In this study, rat skeletal myoblasts were studied by dividing myoblasts into mature skeletal myoblasts. Rat skeletal myoblasts culture are more convenient relative to mouse C2C12 skeletal muscle cells. The purpose of this study was to explore the potential mechanism of improving IR and reducing diabetes and its complications by investigating the effect of LR on lipotoxicity-induced changes in the expression of FAT/CD36, FABP4, GRP78, and GLUT4 in rat skeletal muscle L6 myoblasts.

## 2. Materials and Methods

### 2.1. Materials

Rat L6 myoblast cells were donated by the Hebei General Hospital (Shijiazhuang, Hebei, China). Minimum essential medium (MEM) was purchased from Thermo Fisher (Waltham MA, USA); LR was supplied by Novo Nord (Bagsværd, Denmark); 10% fetal bovine serum (FBS) and trypsin-EDTA were purchased from Biological Industries (Beit HaEmek, Israel); PA and skimmed bovine serum albumin (BSA) were purchased from Sigma Aldrich (Shanghai) Trading Co. (China); FAT/CD36 and FABP4 antibodies were purchased from Abcam (Cambridge, UK); and GRP78, GLUT4, and glyceraldehyde 3-phosphate dehydrogenase (GAPDH) antibodies were purchased from GeneTex (Irvine CA, USA).

### 2.2. Cell Culture

L6 cells were cultured in 25 cm^2^ culture bottles containing MEM supplemented with 10% (*v*/*v*) FBS in a 5% CO_2_ atmosphere at 37°C. When the cell growth was close to covering the bottom of the bottle, the cells were digested in 0.25% trypsin-EDTA and transferred into 6-well plates. When cell density reached 60–70%, differentiation was induced by replacing the culture medium with MEM containing 1% FBS, and cells were cultured for 6 days in a 5% CO_2_ atmosphere at 37°C.

### 2.3. Palmitic Acid-Supplemented Culture Medium

By reference to research by Huang et al., we used 0.6 nmol/L for Treatment with Palmitic Acid [10]. The PA treatment solution was prepared by dissolving PA in 75% ethanol at 100 mmol/L. This solution was diluted 1 : 5 in 10% BSA, phacoemulsified, placed at 55°C for 20 min, filtered through a 45 *μ*m membrane, cooled to 37°C diluted in MEM to a final concentration of 0.6 mmol/L, and incubated for 24 h prior to commencing the experiment at this temperature [10].

### 2.4. Interventions

Differentiated cells were randomly divided into five experimental groups: control group, high-fat group, and three LR-intervention groups. Interventions were conducted for 24 h. The control group was cultured in MEM. The high-fat and LR-intervention groups were cultured in MEM supplemented with PA at 0.6 mmol/L. The medium of the LR-intervention groups was additionally supplemented with LR at 10 nmol/L (10LR), 100 nmol/L (100LR), or 1000 nmol/L (1000LR) [10–12].

### 2.5. Cell Viability

Cell viability was measured using a Cell Counting Kit-8 (CCK-8; Sigma Aldrich, Shanghai). Cell suspensions were adjusted to a density of 9 × 10^4^ cells/mL and transferred into wells of 96-well plates, 100 *μ*L per well, and incubated at 37°C for 48 h. Following interventions, 10 *μ*L of CCK-8 reagent was added to each well, and the plates were placed at 37°C for 1 h. The optical density (OD) was measured at 450 nm using a microplate reader (BD680 type automatic microplate reader Bio Rad company, USA). Stimulation index SI = (OD value of each stimulated well − OD value of medium)/(OD value of unstimulated well − OD value of medium).

### 2.6. Lipid Deposition

Lipid deposition was measured using an Oil Red O Staining Kit (Solarbio Life Sciences; Beijing, China). Cells were washed twice with PBS, fixed with 4% paraformaldehyde for 15 min; washed twice with PBS, pretreated with 60% isopropanol for 20 s, and washed twice with PBS after 30 min shading, and separated with 60% isopropanol for 5 s, and then washed twice with PBS. After the staining area was calibrated and normalized using Image J image processing software, the data were counted. Stained cells were photographed with an inverted microscope (Tu Ming Company, Shanghai, China) within 2 h of staining.

### 2.7. Triglyceride Content

Intracellular triglyceride content was quantified using a Triglyceride (TG) Content Assay Kit (Solarbio Life Sciences; Beijing, China) according to the manufacturer's protocol.

### 2.8. mRNA Expression

Total RNA was extracted from treated cells using kit (TIANGEN Biochemical Technology Company, Beijing, China) according to the manufacturer's protocol. The gene expression levels were measured by reverse transcription quantitative real-time PCR using Loading Buffer PCR Master Mix (TIANGEN Biochemical Technology Company). Primers were synthesized by GeneCopoeia (Rockville MD, USA; Table 1). Cycling conditions were as follows: 95°C for 10 min; 40 cycles of 60°C for 20 s, and 70°C for 10 s. Amplification was carried out on a Cobasz 480 (Roche Company, Switzerland) to confirm the amplification curve, dissolution curve, and Ct value using 2-*ΔΔ*Ct to calculate the mRNA expression of the target gene.

### 2.9. Protein Expression

Protein expression was quantified using western blotting with GAPDH as the internal reference. Protein samples were separated by polyacrylamide gel electrophoresis, transferred to a nitrocellulose membrane, and the membranes blocked with milk for 1 h and probed overnight at 4°C with primary antibodies. The antibodies used were as follows:

The immunoreactive bands were separated by polyacrylamide gel electrophoresis, transferred to a nitrocellulose membrane, and blocking repeated. Membranes were washed, probed overnight at 4°C with sheep anti-rabbit (GRP78, FAT/CD36, FABP4, and GLUT4) or sheep anti-rat (GAPDH) secondary antibodies. Immunoreactive bands were detected using chemiluminescence reagent (TIANGEN Biochemical Technology Company) according to the manufacturer's instructions. Protein expression was quantified using an enzyme labeling instrument (BD 680 automatic microplate reader) at 570 nm.

### 2.10. Statistical Analyses

All data are expressed as means ± standard deviation (*n* = 3). Statistical significance was assessed using Fisher LSD *t*-tests, and single factor ANOVA; correlations were determined using linear correlation. SPSS 20.0 (IBM Corp; Armonk NY, USA) was used for statistical analysis, and GraphPad Prism 6.01 (GraphPad Software; La Jolla CA, USA) was used for graphic analysis. Statistical significance was set at *P* < 0.05.

## 3. Results

### 3.1. Cell Viability

Cell viability was reduced in the high fatgroup (average SI = 2.70) relative to the control group (average SI = 2.17) (2.17 ± 0.06 vs. 2.70 ± 0.08, *P* < 0.01), of the 10LR group (average SI = 2.63) did not differ from the high-fat group (2.63 ± 0.12 vs. 2.70 ± 0.08, *P* > 0.05), but both the 100LR (average SI = 2.64) and 1000LR (average SI = 2.50) groups had higher cell viability (*P* < 0.01) [10, 11]. Cell viability highest in the 100LR group (*P* < 0.05).

### 3.2. Triglyceride Content

The TG content was higher in the high-fat group relative to the control group (0.13 ± 0.02 vs. 0.04 ± 0.01, *P* < 0.01). The 10LR group did not differ from the high-fat group, but both the 100LR (0.09 ± 0.01) and 1000LR (0.06 ± 0.02) groups had a lower TG content than the high-fat group (*P* < 0.01).

### 3.3. Lipid Deposition

**Figure 1:**
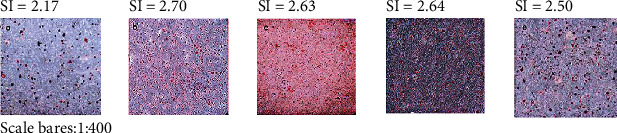
Oil red O staining of myoblast cells treated with palmitic acid (PA) + different concentrations of liraglutide (LR). (a) control group. (b) high-fat group (0.6 mmol/L PA). (c) 10LR group (0.6 mmol/L PA + 10 nmol/L LR). (d) 100LR group (0.6 mmol/L PA + 100 nmol/L LR). (e) 1000LR group (0.6 mmol/L PA + 1000 nmol/L LR).

Lipid deposition was lower in all three LR-intervention groups relative to the control group and decreased with increasing LR concentration (Figure 1).

### 3.4. mRNA Expression

**Figure 2:**
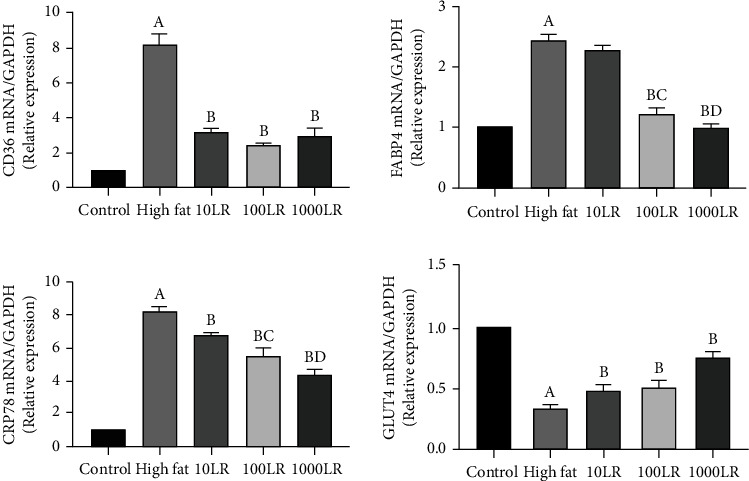
The effect of liraglutide at 0, 10, 100, or 1000 nmol/L on the levels of GRP78, FAT/CD36, FABP4, and GLUT4 mRNA in myoblasts with palmitic acid-induced lipotoxicity. Data are presented as means ± standard deviation (*n* = 3). Bars with different letters are significantly different (*P* < 0.01). Abbreviations: GRP78: glucose regulatory protein 78; CD36: fatty acid transfer protein; FABP4: fatty acid binding protein, GLUT4: glucose transporter 4; LR: liraglutide; PA: palmitic acid.

Level of GRP78, FAT/CD36, and FABP4 mRNA were higher, and GLUT4 mRNA lower in the high-fat group relative to the control group (*P* < 0.01). Levels of FAT/CD36 and GRP78 mRNA were lower and GLUT4 mRNA higher in all three LR-intervention groups relative to the high-fat group. FABP4 mRNA in the 100LR and 1000LR groups were also lower compared with the high-fat group (Table 2, Figure 2).

### 3.5. Protein Expression

**Figure 3:**
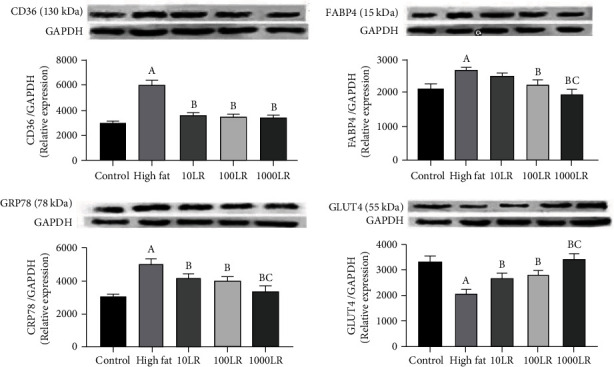
The effect of liraglutide at 0, 10, 100, or 1000 nmol/L on the levels of GRP78, FAT/CD36, FABP4, and GLUT4 proteins in myoblasts with palmitic acid-induced lipotoxicity. Data are presented as means ± standard deviation (*n* = 3). Bars with different letters are significantly different (*P* < 0.01). Abbreviations: GRP78: glucose regulatory protein 78; CD36: fatty acid transfer protein; FABP4: fatty acid binding protein, GLUT4: glucose transporter 4; LR: liraglutide; PA: palmitic acid.

Levels of GRP78, FAT/CD36, and FABP4 proteins were higher, and that of GLUT4 protein lower in the high fat group relative to the control group. Levels of FAT/CD36 and GRP78 protein were lower and GLUT4 protein higher in all three LR-intervention groups compared with the high-fat In the 100LR and 1000LR groups, levels of FABP4 protein were also lower compared with the high-fat group (Table 3, Figure 3).

### 3.6. Correlations

Intracellular TG content was positively correlated with levels of GRP78 protein, FAT/CD36 protein, and FABP4 protein, and negatively correlated with levels of GLUT4 protein (Tables 4 and 5).

## 4. Discussion

At 100 and 1000 nmol/L, LR reversed the effects of lipotoxicity on cell viability and levels of FABP4 mRNA/protein. At 10, 100, and 100 nmol/L, LR reversed lipotoxicity-induced effects on levels of FAT/CD36, GRP78, and GLUT4 mRNA/protein. LR also reversed PA-induced lipid deposition in a concentration-dependent manner. Intracellular TG content was positively correlated with expression of FAT/CD36, FABP4, and GRP78 proteins, and negatively correlated with expression of GLUT4 protein. LR reversed PA-induced lipotoxicity and endoplasmic reticulum stress by decreasing the expression of proteins that control fatty acid transport and intracellular lipid deposition and increasing the expression of proteins responsible glucose transport.

In this study, PA-induced lipotoxicity resulted in decreased cell viability, lipid deposition, and increased intracellular TG content in L6 myoblasts. Likewise, a previous study found that PA-induced lipotoxicity led to intracellular TG accumulation and decreased hepatocyte activity [12], and affected the function and activity of osteoblasts in vitro [13]. In the results of CCK-8, we found that the cell viability of the 100LR group was higher than that of the 1000LR group. Recent studies have shown that LR is hepatotoxic and can affect cell proliferation [14]. We speculate that the reason for this result may be related to the cytotoxicity of LR, but other experiments are still needed to verify.

As one of the main target tissues of insulin, skeletal muscle plays an important role in the development of IR. As an observation index of IR, GLUT4 also plays an important role in skeletal muscle. In this experiment, the expression of GLUT4 mRNA and protein decreased in L6 myoblasts incubated with PA. The addition of LR increased the expression of GLUT4 mRNA and protein in a concentration-dependent manner, which was consistent with Ji et al. [15] who found that LR upregulated the expression of GLUT4 and improved IR in type 2 diabetes skeletal muscle through PTP1B and PI3K/Akt2 signal pathways. The content of TG in cells was negatively correlated with the expression of GLUT4 protein. These results suggest that LR can effectively improve the IR state of L6 myoblasts induced by PA, which may be related to the improvement of intracellular TG content.

In this study, skeletal muscle myoblasts are used as the research object, and the research is carried out by differentiating myoblasts into mature skeletal muscle cells, because mature skeletal muscle cells lose their ability to divide and are difficult to culture [16–19]. In skeletal muscle cells, FAT/CD36 is the main membrane protein that promotes fatty acid transport, a process related to the metabolic stress signal cascade [9]. The binding of FABP4 to fatty acids in the cytoplasm regulates the rate of lipid uptake throughout the transmembrane transport system [16]. In a previous study [17], LR was found to reduce the TG and long-chain fatty acyl-CoA content in skeletal muscle cells of high-fat-fed rats in a concentration-dependent manner. In this study, LR reversed the increases in intracellular lipid deposition and FAT/CD36 and FABP4 mRNA and protein expression induced by PA. Correlation analysis showed that intracellular TG content was positively correlated with the expression of FAT/CD36 and FABP4 proteins. This is consistent with the results of Li et al. [20] who found that LR reduced lipid deposition in rat heart cells by reducing FAT/CD36 expression. Previous experiments have found that the cAMP/PKA/CREB pathway affects lipolysis and fatty acid oxidation of adipose tissue by changing the expression of FAT/CD36 and FABP4 [21]. We speculate that LR intervention can reduce cellular lipid deposition by downregulating the expression of FAT/CD36 and FABP4; however, the specific mechanism requires further investigation.

Studies have shown that ERS may be the main way to induce IR [22–24]; GRP78 is an index protein reflecting the function of built-in reticulum. A previous study showed that LR can improve lipid metabolism and liver lipid deposition in high-fat-fed rats in a concentration-dependent manner, and the mechanism of reducing IR, may be related to the inhibition of GRP78/JNK pathway in the liver [25, 26]. In this experiment, LR reversed the increase in GRP78 mRNA and protein expression induced by PA in L6 cells, and that expression of GRP78 protein was positively correlated with intracellular TG content. The improved lipid metabolism conferred by LR may be related to reduce ERS. In the other two studies, LR also improves lipid metabolism by regulating other pathways, which is consistent with this study [27, 28].

Li et al. [29] studies have a conclusion similar to the study by exploring the IRS-Serine Channel. Compared with the research of Li et al., our research focuses on FAT/cd36, fabp4, GRP78, and other factors caused by palm acid. Lipid metabolism, IR, and type 2 diabetes have been the focus of research worldwide, but the specific molecular mechanisms remain unclear [29, 30]. This study provides insight into the mechanism of LR to reduce cellular lipid deposition and improve IR. LR may improve IR by reducing ERS through regulation of fatty acid transport reduce lipid deposition, increase the expression of GLUT-4. The findings in the study have important theoretical significance and prospective application for further discovery of the molecular mechanism of LR in the treatment of type 2 diabetes and the subsequent development of new drugs to treat the disease. Future studies that investigate the effects of blocking or activating signal pathways investigated in this study will further progress our understanding of the mechanism involved in IR.

In summary, LR reduced the ERS response and intracellular lipid deposition, improved lipid metabolism, and downregulated the expression of GRP78, FAT/CD36, and FABP4 mRNA and protein in muscle cells with PA-induced lipotoxicity. In addition, LR improved the state of IR by regulating the expression of GLUT4 in the insulin transduction pathway. This study gives insight into how LR improves IR and provides theoretical support for its clinical application.

## 5. Limitation

There are also some limitations in our experiments. Some experiments can be added as proof of the result: (1) cell viability assays have been performed but apoptosis assays like Annexin V assay or Hoechst assay can be performed to visualize the percent of cells undergoing apoptosis with palmitic acid and liraglutide treatment [31–33]. (2) ORO staining has been performed to measure the lipid deposition but semiquantification of the lipid droplets in ORO staining or counting the ORO positive cells in each group can be performed to determine the differences between each group [34]. (3) Lipid deposition could be verified at mRNA and protein levels using perilipin as a marker to provide additional support to the results [35].

## Figures and Tables

**Table 1:** Primers used in quantitative RT-PCR.

Gene	Forward primer	Reverse primer	Gene accession number
*CD36*	AACCCAGAGGAAGTGGCAAG	GACAGTGAAGGCTCAAAGATGG	NC_051339.1
*GRP78*	AACCCCAGATGAGGCTTGTAGCA	ACCATCAAGCAGAAACCAGGTCAC	NC_036026.1
*FABP4*	AAGTCAAGAGCACCATAACC	GATACATTCCACCACCAAAACT	NC_051337.1
*GLUT4*	GATGCCGTCGGGTTTCCAGCA	TGAGGGTGCCTTGTGGGATGG	NC_051345.1
*GAPDH*	GGCACAGTCAAGGCTTGAAGAG	ATGGTTGGTGAAGAGAGCGCCAGTA	NC_051339.1

**Table 2:** mRNA expression of each group factor ($\bar{x}\pm s$).

Group	GRP78 mRNA	FABP4 mRNA	FAT/CD36 mRNA	GLUT4 mRNA
Contol group	1 ± 0.00	1 ± 0.00	1 ± 0.00	1 ± 0.00
High fat group	8.15 ± 0.35^a^	2.46 ± 0.08^a^	8.29 ± 0.52^a^	0.34 ± 0.03^a^
10LR group	6.83 ± 0.11^b^	2.28 ± 0.06	3.23 ± 0.11^b^	0.47 ± 0.06^b^
100LR group	5.52 ± 0.48^bc^	1.24 ± 0.06^bc^	2.42 ± 0.11^b^	0.50 ± 0.04^b^
1000LR group	4.36 ± 0.32^bd^	1.00 ± 0.04^bd^	2.88 ± 0.55^b^	0.75 ± 0.04^b^

^a^ *P* < 0.01 vs. control group; ^b^*P* < 0.01 vs. high-fat group; ^c^*P* < 0.01 vs. 10LR group; ^d^*P* < 0.01 vs. 100LR group.

**Table 3:** Protein expression of each group factor ($\bar{x}\pm s$).

Group	GRP78	FABP4	FAT/CD36	GLUT4
Control group	2984.09 ± 222.64	2107.22 ± 179.32	2994.59 ± 107.29	3346.60 ± 229.29
High fat group	4963.98 ± 277.29^a^	2676.07 ± 100.64^a^	6083.20 ± 284.70^a^	2137.79 ± 118.75^a^
10LR group	4169.24 ± 226.99^b^	2503.84 ± 91.67	3644.59 ± 223.85^b^	2693.05 ± 206.32^b^
100LR group	3943.69 ± 297.23^b^	2235.84 ± 159.18^b^	3457.17 ± 272.31^b^	2792.29 ± 225.72^b^
1000LR group	3338.13 ± 333.15^bc^	1978.85 ± 124.24^bc^	3372.00 ± 219.84^b^	3443.71 ± 191.89^bc^

^a^ *P* < 0.01 vs. control group; ^b^*P* < 0.01 vs. high-fat group; ^c^*P* < 0.01 vs. 10LR group.

**Table 4:** Correlations between intracellular triglyceride (TG) content and levels of four proteins.

	GRP78		FABP4		FAT/CD36		GLUT4	
TG	r	p	r	p	r	p	r	p
	0.975	0.002^∗∗^	0.872	0.027^∗^	0.975	0.002^∗∗^	-0.872	0.027^∗^

^∗^ *P* < 0.05; ^∗∗^*P* < 0.01.

**Table 5:** 

Antibody name	Production company
GRP78 (1∶1000)	Epitomics corporation, USA
FAT/CD36 (1∶1000)	Wuhan HealthCare Biotechnology Co., Ltd.
FABP4 (1∶1000)	Abcam corporation, USA
GAPDH (1∶5000)	Boster co., ltd., China
Sheep anti-rabbit IgG secondary antibody (1∶5000)	KPL corporation, USA
Goat anti-rabbit secondary antibody (1∶5000)	KPL corporation, USA

## Data Availability

All data are fully available without restriction.
